# Improving the accuracy of multi breath-hold diffusion tensor MRI tractography of the heart using dynamic motioncorrection

**DOI:** 10.1186/1532-429X-15-S1-O81

**Published:** 2013-01-30

**Authors:** Choukri Mekkaoui, Sonia Nielles-Vallespin, Marcel P Jackowski, Peter D Gatehouse, Dudley J Pennell, David N Firmin, David E Sosnovik

**Affiliations:** 1 Radiology, Harvard Medical School - Massachusetts General Hospital, Boston, MA, USA

## Background

Diffusion Tensor MRI (DTI) tractography of the human heart can be performed *in vivo*, but requires multiple breatholds per slice to achieve adequate SNR [1,2]. The physiological noise inherent to multiple breatholds results in a diffusion-encoded volume in which the data vary as a function of space and time. This may hinder the assessment of diffusion based-indices in the heart and accurate tractography of myofiber architecture. In this study, we investigate the effect of a novel automated motion correction method on *in vivo* DTI of the heart.

## Methods

**Figure 1:**
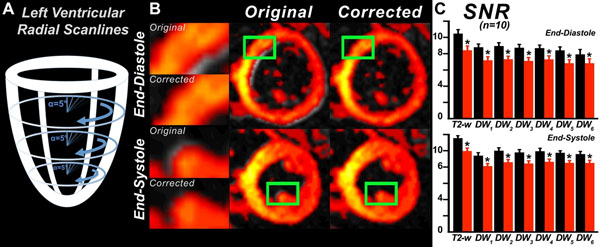
Motion correction of images in a diffusion-encoded 3D volume. (A) The diffusion encoded volume consisted of a slice at the midventricular level, a more basal slice and a more apical slice. Radial scanlines with an angular resolution of 5 degrees were acquired and used for matching frames at each level. (B) Overlay of two short-axis slices, one depicted in grayscale and the other in a hot colormap, before (original) and after motion correction. Alignment of the images and their SNR (C) is significantly improved (p<0.01, Mann-Whitney) by motion correction (black bars = corrected; red bars = original).

DTI of 10 normal volunteers was performed on a 3T clinical scanner (Skyra, Siemens) with the following parameters: 6 diffusion-encoding directions, b=350 s/mm^2^, fat saturation, TR/TE=1100/23 ms, BW=2442 Hz/pixel, spatial resolution=2.7x2.7x8 mm^3^, 8 averages. This required 24 separate breatholds for a 3-slice diffusion-encoded volume. On a chosen reference frame, n radial scanlines (Figure 1A) starting at the LV-RV junction and crossing at the center of the LV cavity were defined as the registration axes. Motion was estimated by matching the intensity gradient profiles of corresponding radial scanlines between frames and at all 3 slice levels. A rigid registration described by a set of translations and rotations was then determined between pairs of images, iteratively (Figure 1B). Convergence was achieved upon reaching a global minimum of the quadratic error at each level. The signal-noise-ratio (SNR) was calculated at each pixel **r** with a given number of averages (repetitions) t, such that SNR_t_(**r**) = MEAN_t_(**r**) / SD_t_(**r**) [3]. SNR, with and without motion correction, was measured in each voxel at end-diastole and end-systole. Fiber tracking was performed with a 4^th^ order Runge-Kutta approach [4].

## Results

**Figure 2:**
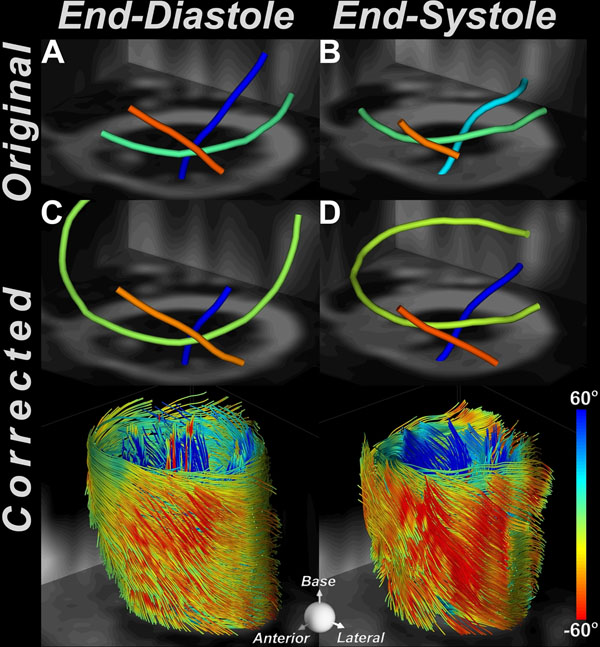
DTI-tractography *in vivo* with and without motion correction. Motion correction frequently increased the length of the tracked fibers. This can be best appreciated through comparison of the midmyocardial fibers (green-yellow) without motion correction (2A, 2C) and with motion correction (2B, 2D). Motion correction also rectified the orientation of some of the myofibers (endocardial fibers in systole, 2B *vs*. 2D).

At end-diastole, an increase of 24% was observed in the SNR of the T2-weighted images, and an average SNR increase of 21% was seen in the diffusion-weighted images (Figure 1C). At end-systole, a relatively uniform SNR increase of 15% was seen with motion correction for diffusion-free and diffusion-weighted images. Figure 2 shows tractograms in the lateral wall of the left ventricle at end-diastole and end-systole. Motion correction frequently increased the fiber track lengths, consistent with the observed increase in SNR, and in some cases also rectified their orientation (see endocardial fibers at end-systole, Figure 2B vs. 2D).

## Conclusions

DTI tractography can be performed *in vivo* over multiple breatholds, but is affected by physiological noise. Here, we show that motion correction over space and time can reduce the noise produced by multiple breatholds while increasing the quality of the resulting tractograms. This approach paves the way for the use of free breathing navigator-based DTI for high-resolution tractography of the heart.

## Funding

R01HL093038.
